# An upgraded rotatable sphincterotome enhances bile duct cannulation via balloon endoscopy-assisted endoscopic retrograde cholangiopancreatography

**DOI:** 10.1055/a-2549-2747

**Published:** 2025-03-28

**Authors:** Yuya Takenaka, Katsuyuki Miyabe, Toshitaka Mori, Naoki Atsuta, Yasuki Hori, Tomonori Yamada, Kazuki Hayashi

**Affiliations:** 1 13789 Department of Gastroenterology, Japanese Red Cross Aichi Medical Center Nagoya Daini Hospital, Nagoya, Japan; 2 Department of Gastroenterology and Metabolism, Nagoya City University Graduate School of Medical Sciences, Nagoya, Japan; 3 36975 Department of Gastroenterology, Nagoya City University East Medical Center, Nagoya, Japan

Bile duct cannulation via balloon endoscopy-assisted endoscopic retrograde cholangiopancreatography (ERCP) can be challenging, particularly in complex anatomical scenarios 1 2 . This case report emphasizes the clinical application of a novel rotatable sphincterotome in a 75-year-old man who presented to a local clinic with a 1-week history of bilirubinuria. The patient had a history of gastric cancer and had undergone a distal gastrectomy with Roux-en-Y reconstruction 6 years previously. Laboratory tests revealed elevated liver enzymes, prompting a referral to our hospital. Contrast-enhanced computed tomography and magnetic resonance cholangiopancreatography revealed mild common bile duct wall thickening and stricture with upstream biliary dilation ( Fig. 1 , Fig. 2 ), which was eventually diagnosed as recurrent gastric cancer 6 months after ERCP.

**Fig. 1:**
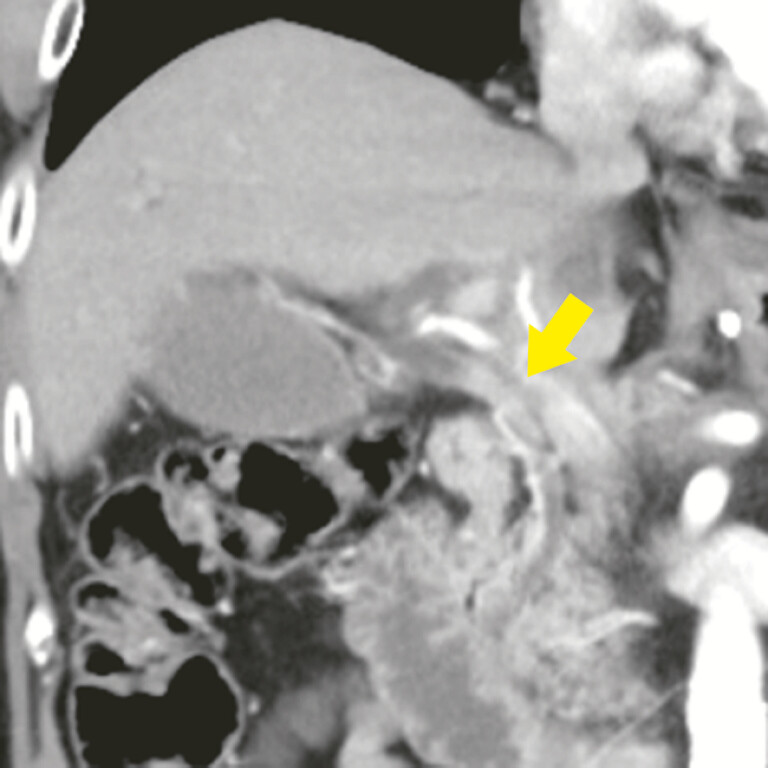
Contrast-enhanced computed tomography in a patient with a history of gastric cancer treated with distal gastrectomy and Roux-en-Y reconstruction revealed mild thickening and stricture (arrow) of the common bile duct wall and upstream biliary dilation.

**Fig. 2:**
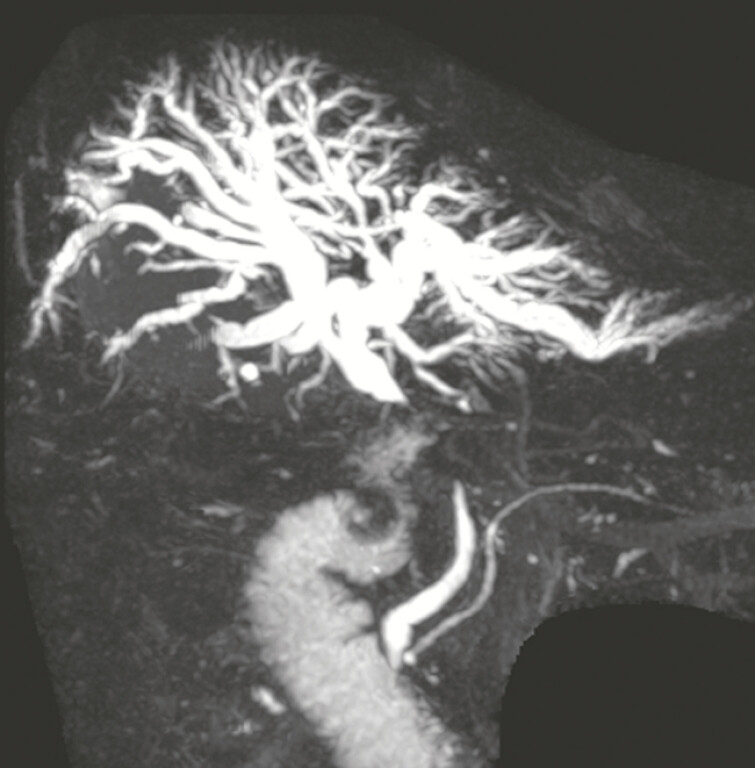
Magnetic resonance cholangiopancreatography revealed a stricture of the common bile duct with upstream biliary dilation.

A double-balloon endoscope was used to access the papilla. However, significant challenges prevented successful bile duct cannulation. Retroflex position, a technique often used to facilitate cannulation 3 , was unsuccessful due to the narrow duodenal lumen. Furthermore, conventional sphincterotomy failed as the instrument could not rotate adequately under balloon-assisted endoscopy, and the curvature of the knife did not align with the bile duct axis. Subsequently, a novel, upgraded sphincterotome (Aimingtome; Asahi Intecc Co., Ltd., Seto, Japan) was used ( Fig. 3 ) 4 . This device features a more rotatable and flexible tip, which enabled guidewire insertion into the duodenal papilla ( Video 1 ). The guidewire was then successfully advanced into the main pancreatic duct, facilitating bile duct cannulation via the pancreatic duct guidewire technique. Endoscopic sphincterotomy was performed using the same sphincterotome ( Fig. 4 ), followed by the placement of a biliary plastic stent ( Fig. 5 ). The patient was discharged 3 days after the procedure. In cases where frontal visualization of the papilla using balloon endoscopy-assisted ERCP is challenging, the use of a novel rotatable sphincterotome can effectively facilitate bile duct cannulation and subsequent endoscopic sphincterotomy.

**Fig. 3:**
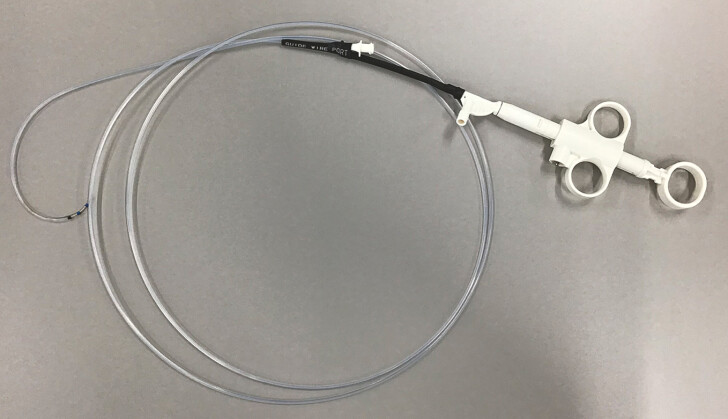
Macroscopic overview of the novel sphincterotome. Source: Asahi Intecc, Seto, Japan.

An upgraded rotatable sphincterotome successfully facilitated bile duct cannulation using balloon endoscopy-assisted endoscopic retrograde cholangiopancreatography. Source for sphincterotome: Asahi Intecc, Seto, Japan.Video 1

**Fig. 4:**
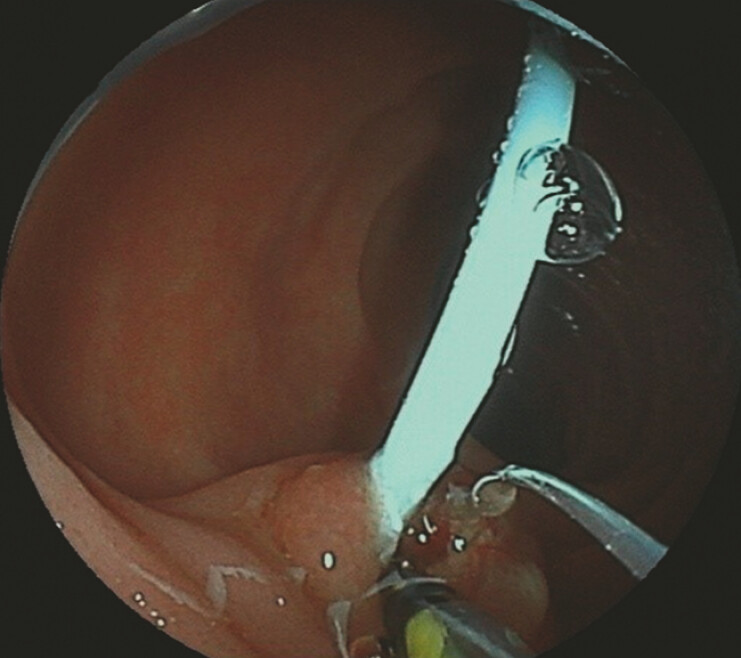
Endoscopic sphincterotomy using the novel sphincterotome. Compared to a conventional sphincterotome, it allows 360° rotation and greater backward flexibility.

**Fig. 5:**
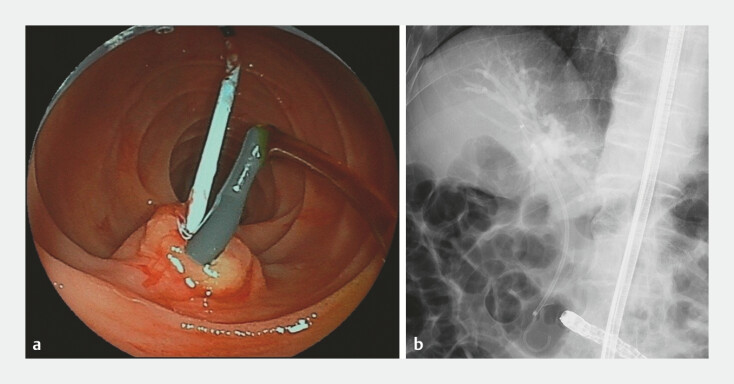
Biliary and pancreatic stents placed in the common bile duct and main pancreatic duct: **a** endoscopic view; **b** radiographic image

Endoscopy_UCTN_Code_TTT_1AR_2AC

## Notes

Endoscopy E-Videos https://eref.thieme.de/e-videos
*E-Videos* is an open access online section of the journal *Endoscopy* , reporting on interesting cases and new techniques in gastroenterological endoscopy. All papers include a high-quality video and are published with a Creative Commons CC-BY license. Endoscopy E-Videos qualify for HINARI discounts and waivers and eligibility is automatically checked during the submission process. We grant 100% waivers to articles whose corresponding authors are based in Group A countries and 50% waivers to those who are based in Group B countries as classified by Research4Life (see: https://www.research4life.org/access/eligibility/ ). This section has its own submission website at https://mc.manuscriptcentral.com/e-videos .
